# The panoramic picture of pepsinogen gene family with pan‐cancer

**DOI:** 10.1002/cam4.3489

**Published:** 2020-10-17

**Authors:** Shixuan Shen, Hao Li, Jingwei Liu, Liping Sun, Yuan Yuan

**Affiliations:** ^1^ Tumor Etiology and Screening Department of Cancer Institute and General Surgery The First Hospital of China Medical University Shenyang China; ^2^ Key Laboratory of Cancer Etiology and Prevention in Liaoning Education Department The First Hospital of China Medical University Shenyang China; ^3^ Key Laboratory of GI Cancer Etiology and Prevention in Liaoning Province The First Hospital of China Medical University Shenyang China

**Keywords:** copy number, expression, immune cell, mutation, pan‐cancer, pathways, pepsinogen, prognosis, risk

## Abstract

**Background:**

It is well known that pepsinogen (PGs), as an important precursor of pepsin performing digestive function, has a good correlation with the occurrence and development of gastric cancer and it is also known that ectopic PGs expression is related to the prognosis of some cancers. However, the panoramic picture of pepsinogen gene family in human cancer is not clear. This study focused on elucidating the expression profile, activated pathway, immune cells infiltration, mutation, and copy number variation of PGs and their potential role in human cancer.

**Method:**

Based on the next generation sequence data from TCGA, Oncomine, and CCLE, the molecular changes and clinical correlation of PGs in 33 tumor types were analyzed systematically by R language, including the expression, mutation, and copy number variation of PGs and their correlation with cancer‐related signal transduction pathway, immune cell infiltration, and prognostic potential in different cancers.

**Results:**

PGs expression profiles appear different in 33 tumors. The transcriptional expression of PGs was detected in 16 of all 33 tumors. PGC was highly expressed in cholangiocarcinoma, colon adenocarcinoma, rectum adenocarcinoma, uterine corpus endometrial carcinoma, bladder urothelial carcinoma and breast cancer, while decreased in stomach adenocarcinoma, kidney renal clear cell carcinoma, prostate adenocarcinoma, lung squamous cell carcinoma, and esophageal carcinoma. PGA3, PGA4, and PGA5 were expressed in most normal tissues, but decreased in cancer tissues. PGs expression was significantly related to the activation or inhibition of many signal transduction pathways, in which PGC and PGA5 are more likely to be associated with cancer‐related pathways. PGC participated in 33 regulatory network pathways in pan‐cancer, mainly distributed in stomach adenocarcinoma, esophageal carcinoma, and lung squamous cell carcinoma, respectively. PGC and PGA3 expression were significantly correlated with immune cell infiltration. The results of survival analysis showed that different PGs expression play significantly different prognostic roles in different cancers. PGC was correlated with poor survival in brain lower grade glioma, skin cutaneous melanoma, and higher survival in kidney renal clear cell carcinoma, acute myeloid leukemia, mesothelioma, and uveal melanoma. PGA4 was only associated with higher survival in kidney renal clear cell carcinoma. Genetic variation analysis showed that PGC gene often mutated in uterine corpus endometrial carcinoma and stomach adenocarcinoma had extensive copy number amplification in various tumor types. PGC expression was upregulated with the increase of copy number in cholangiocarcinoma, esophageal carcinoma, and kidney renal papillary cell carcinoma, while in stomach adenocarcinoma, PGC was upregulated regardless of whether the copy number was increased or decreased.

**Conclusions:**

PGs was expressed unevenly in a variety of cancer tissues and was related to many carcinogenic pathways and involved in the immune regulation. PGC participated in 33 regulatory pathways in human cancer. Different PGs expression play significantly different prognostic roles in different cancers. The variation of copy number of PGC gene could affect the PGC expression. These findings suggested that PGs, especially PGC have characteristic of broad‐spectrum expression in multiple cancers rather than being confined to the gastric mucosa, which may made PGs be useful biomarkers for prediction/diagnosis/prognosis and effective targets for treatment in human cancer.

## Graphical abstract

Based on the next generation sequence data from TCGA and CCLE, the molecular changes and clinical correlation of PGs in 33 tumor types were analyzed systematically including the expression profiles, mutation and copy number variation of PGs and their correlation with cancer‐related signal transduction pathway, immune cell infiltration and prognostic potential in different cancers. PGs was expressed unevenly in a variety of cancer tissues and was related to many carcinogenic pathways and involved in the immune regulation.Different PGs expression play significantly different prognostic roles in different cancers. The variation of copy number of PGC gene could affect the PGC expression.

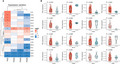

## 1 INTRODUCTION

The Cancer Genome Atlas (TCGA) has been well known and widely used.^1^ As its final work, the Pan‐Cancer Atlas is getting to know well, which made a multigroup integrated analysis in the aspects of Cell Origin Patterns, Oncogenic Processes, and Tumor Signaling Pathways (http://www.cell.com/pb‐assets/consortium/pancanceratlas/pancani3/index.html) and provides a referential idea for us to study cancer from the point of view of broad‐spectrum molecular characteristics. Through pan‐cancer analysis, we learned that tumors that occurred in different organs with the same histomorphological type, such as squamous cell carcinoma of the head, neck, lungs, esophagus, bladder, and cervix, had strong molecular similarity. There are also molecular similarities in cancers with similar anatomical structure but different location, such as gastric cancer, colon cancer, and rectal cancer.^2^ On the contrary, some cancers occur in the same organ, but may belong to completely different molecular subtypes, such as kidney cancer.^3^ The findings mentioned above suggest that a comprehensive longitudinal analysis of the panoramic picture of certain molecular events in a variety of tumors can identify the mutual molecular characteristics among many kinds of human cancers, which can provide new insights into the clinical feasibility of comprehensive cancer therapy and the development of new targeted and combined therapies.

Pepsinogen gene family (PGs) are important precursors of pepsin that performs digestive function in stomach, and they belong to the family of aspartate proteolytic enzymes.^4^ According to the immunological and biochemical characteristics, PGs can be divided into two types: PGA and PGC. Among them, PGA can be further divided into three subtypes of PGA3, PGA4, and PGA5. PGs are mainly synthesized by the chief cells of gastric mucosa, stored in zymogen granules at rest, secreted into the gastric cavity once receiving physiological or external chemical signals, and activated into pepsin in the acidic environment of gastric juice. It is traditionally believed that PGs are the final products of the differentiation and maturation of gastric mucosal cells and the sign of the gradual maturity of digestive function. Previous studies have confirmed that the expression of PGs have a good correlation with the occurrence of gastric cancer, which could be used as biomarkers for screening gastric cancer and its precursors.^5^, ^6^ In addition to in situ expression in gastrointestinal tissues, PGs is also expressed in a few tissues outside the gastrointestinal tract, such as PGC in normal seminal vesicles and lung type II epithelial cells.^7^ Interestingly, recent studies have found that PGC can be ectopic expressed in tumor tissues that were not expressed initially, such as prostate cancer, breast cancer, ovarian cancer, endometrial cancer, pancreatic cancer, kidney cancer, bladder cancer, eyelid basal cell carcinoma, squamous cell carcinoma, melanoma, and so on.^8^, ^9^, ^10^ PGA is expressed in esophageal squamous cell carcinoma.^11^ The results of these studies suggest that PGs may be closely related to many kinds of tumors. At present, the panoramic picture of PGs expression in pan‐cancer is not clear, the genetic variation of PGs own structure, and its internal effect on the expression are not clear, the relationship between multigroup varieties of PGs and clinical phenotypic characteristics of cancer are not clear. In a word, a series of outstanding matters regarding to PG gene family in human cancer remains to be further explored.

In this study, by using the multilevel data from TCGA based Pan‐Cancer Atlas, Oncomine and Cancer Cell Line Encyclopedia (CCLE), we focused on the elucidating expression profile, activated pathway, immune cells infiltration, mutation, and copy number variation of PGs and their prediction/diagnosis/prognosis potential in pan‐cancer.

## 2 MATERIALS AND METHODS

### 2.1 Data collection

#### 2.1.1 TCGA data collection

We totally collected the information of 33 different kinds of tumors in TCGA database (http://cancergenome.nih.gov/), including the information of TPM (Transcripts Per Kilobase Million) expression, mutation, and copy number variation. The clinical information (survival status, stages, grades, and survival time) were download from UCSC XENA (https://xenabrowser.net/).

TCGA data sources include all 33 different tumor types including adrenocortical carcinoma (ACC); bladder urothelial carcinoma (BLCA); breast cancer (BRCA); cervical squamous cell carcinoma and endocervical adenocarcinoma (CESC); cholangiocarcinoma (CHOL); colon adenocarcinoma (COAD); lymphoid neoplasm diffuse large b‐cell lymphoma (DLBC); esophageal carcinoma (ESCA); glioblastoma multiforme (GBM); head and neck squamous carcinoma (HNSC); kidney chromophobe (KICH); kidney renal clear cell carcinoma (KIRC); kidney renal papillary cell carcinoma (KIRP); acute myeloid leukemia (LAML); brain lower grade glioma (LGG); liver hepatocellular carcinoma (LIHC); lung adenocarcinoma (LUAD); lung squamous cell carcinoma (LUSC); mesothelioma (MESO); ovarian serous cystadenocarcinoma (OV); pancreatic adenocarcinoma (PAAD); pheochromocytoma and paraganglioma (PCPG); prostate adenocarcinoma (PRAD); rectum adenocarcinoma (READ); sarcoma (SARC); skin cutaneous melanoma (SKCM); stomach adenocarcinoma (STAD); testicular germ cell tumors (TGCT); thyroid carcinoma (THCA); thymoma (THYM); uterine corpus endometrial carcinoma (UCEC); uterine carcinosarcoma (UCS); uveal melanoma (UVM). The information of tumor and its control group can be found in Table S1.

#### 2.1.2 Oncomine data collection

Oncomine, a cancer microarray database and a Web‐based data mining platform, was used for validation analysis ^12^, in order to facilitate discovery from TCGA.

#### 2.1.3 Proteomics data collection

The protein expression data of PGs gene are derived from the Protein Atlas data set (https://www.proteinatlas.org/) including 21 kinds of cancers and the corresponding normal tissues.

#### 2.1.4 Cellular data collection

CCLE database（https://portals.broadinstitute.org/ccle）was used to identify the PGs expression, mutation, and copy number variation in different cancer cell lines, including all 431 cell lines from six cancer types.

### 2.2 Multidimensional analysis of the expression of PG gene family in pan‐cancer

#### 2.2.1 Analysis of the expression characteristics of PGs in pan‐cancer

Deseq2 package in R was used to identify differentially expressed PGs in each cancer type. The genes with adjusted *p* < 0.05 and at least twofold expression change (| logFC | ≥2) were identified as PGs expression difference. In oncomine analysis, we also selected | logFC | ≥2, *p* < 0.05, and top 10% gene rank as threshold.

CCLE was used to identify the alternation of the expression of PGs in different cancer cell lines.^13^ Kruskal‐Wallis rank test was used to compare the expression of PGs in different types of cancer cell lines.

#### 2.2.2 Analysis of PGs expression related signal transduction pathway in pan‐cancer

Gene set variation analysis (GSVA), which is a nonparametric method to estimate gene set enrichment variation through expression data set samples, was used to calculate the correlation between tumor marker‐related pathways and PGs expression. We calculated the Pearson correlation coefficient (PCC) between the PGs expression and the pathway activity to certify the PGs related to the activation or inhibition of a certain pathway. The regulatory pathway with |PCC| >0.3 and adjusted *p* < 0.05 has been identified as a significantly correlation.

#### 2.2.3 Correlation analysis of PGs expression with immune cell infiltration in pan‐cancer

In order to explore the relationship between PGs and immune cell infiltration, we calculated the Spearman correlation coefficient (SCC) between PGs expression and immune infiltrating cells. The regulatory pathway with |SCC| > 0.3 and adjusted P‐value <0.05 were identified as a significantly correlation.

#### 2.2.4 Correlation analysis of PGs expression with prognosis in pan‐cancer

To explore whether the expression of PGs was associated with the survival of different cancer patients, we divided all cancer patients into two groups according to the median of PGs expression. The survival rates between the two groups were compared by logarithmic rank test. *p* < 0.05 was statistically significant.

### 2.3 Analysis of PGs mutation and copy number variation in pan‐cancer

#### 2.3.1 Analysis PGs mutation in pan‐cancer

The mutation data of PGs were from both TCGA and CCLE database. The mutation frequency of PGs in each cancer tissue and cell lines was defined as the proportion of mutation in the gene.

#### 2.3.2 Analysis PGs copy number variation in pan‐cancer

The Copy number variation (CNV) data of PGs in different cancers and cell lines was download from TCGA and CCLE database. The frequency of CNV in each cancer type and cell lines was calculated as the proportion of CNV amplification and deletion.

### 2.4 Correlation analysis of PGs mutation, copy number variation, and PGs expression

The relationship between PGs mutation, copy number variation, and PGs expression was analyzed by Mann‐Whitney U test in R software.

## 3 RESULTS

### 3.1 PGs expression profile in pan‐cancer

#### 3.1.1 PGs expression at mRNA level

**Figure 1:**
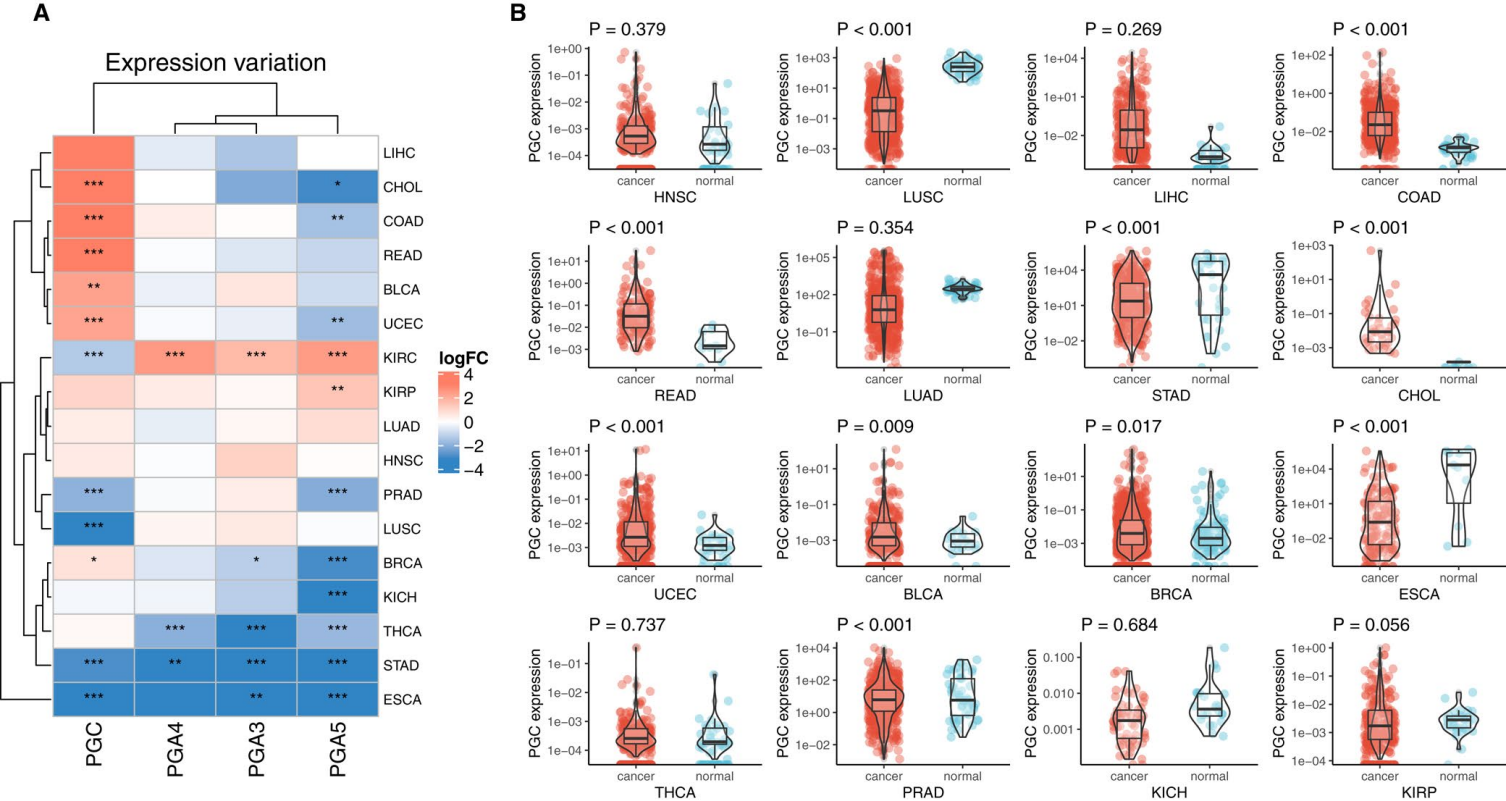
PGs expression profile across different cancer types. A, PGs expression in different cancer and normal tissues. The color in heatmap represents the log2 fold change value between cancer and the normal. The blue color represents the low expression in cancer while the red color represents the high expression in cancer. The * sign represents degree of statistical significance. B, PGC expression in 16 types of cancers between cancer and normal tissues

Using the count data of 33 human tumors covered by TCGA, we analyzed the differential expression of PG family genes including PGC, PGA3, PGA4, and PGA5 in different cancers at the overall level based on continuous variable analysis. The results showed that the PGs expression profiles appear different in 33 tumors. PGC expression was higher in cholangiocarcinoma, colon adenocarcinoma, rectum adenocarcinoma, bladder urothelial carcinoma, uterine corpus endometrial carcinoma, and breast cancer but lower in kidney renal clear cell carcinoma, prostate adenocarcinoma, lung squamous cell carcinoma, stomach adenocarcinoma, and esophageal carcinoma. The expression of PGA3 and PGA4 increased in kidney renal clear cell carcinoma but decreased in thyroid carcinoma and stomach adenocarcinoma. PGA5 expression was higher in kidney renal clear cell carcinoma and kidney renal papillary cell carcinoma, but lower in stomach adenocarcinoma, esophageal carcinoma, cholangiocarcinoma, colon adenocarcinoma, uterine corpus endometrial carcinoma, prostate adenocarcinoma, breast cancer, kidney chromophobe, and thyroid carcinoma (Figure 1A). The differential expression of PGC gene in each cancer was visualized in Figure 1B. In addition, we further compared differential expression between cancer and normal tissues based on categorical variable analysis. When TPM=“median value” was used as the cut‐off value, PGC was highly expressed in hepatocellular carcinoma, colon adenocarcinoma, rectum cancer, and cholangiocarcinoma; and lowly expressed in lung squamous cell carcinoma, lung adenocarcinoma, kidney chromophobe, and kidney renal papillary cell carcinoma. When TPM = 6 was used as the cutoff value, PGC was highly expressed in hepatocellular carcinoma and lowly expressed in lung squamous cell carcinoma, lung adenocarcinoma, and esophageal cancer (Tables S2 and S3).

After that, we selected three well‐studied cancer types including stomach adenocarcinoma, lung squamous cell carcinoma, and colorectal adenocarcinoma among 33 types of tumors from Oncomine database and conducted further analysis, to verify our TCGA findings and predict cancer risk. The results showed that in stomach adenocarcinoma and lung squamous cell carcinoma, high expression of PG is a protective factor, and high expression can reduce the risk of cancer. However, high expression in colorectal adenocarcinoma suggests an increased risk of cancer. These results were consistent with our TCGA findings (Figure S1).

#### 3.1.2 PGs expression at protein level

**Figure 2:**
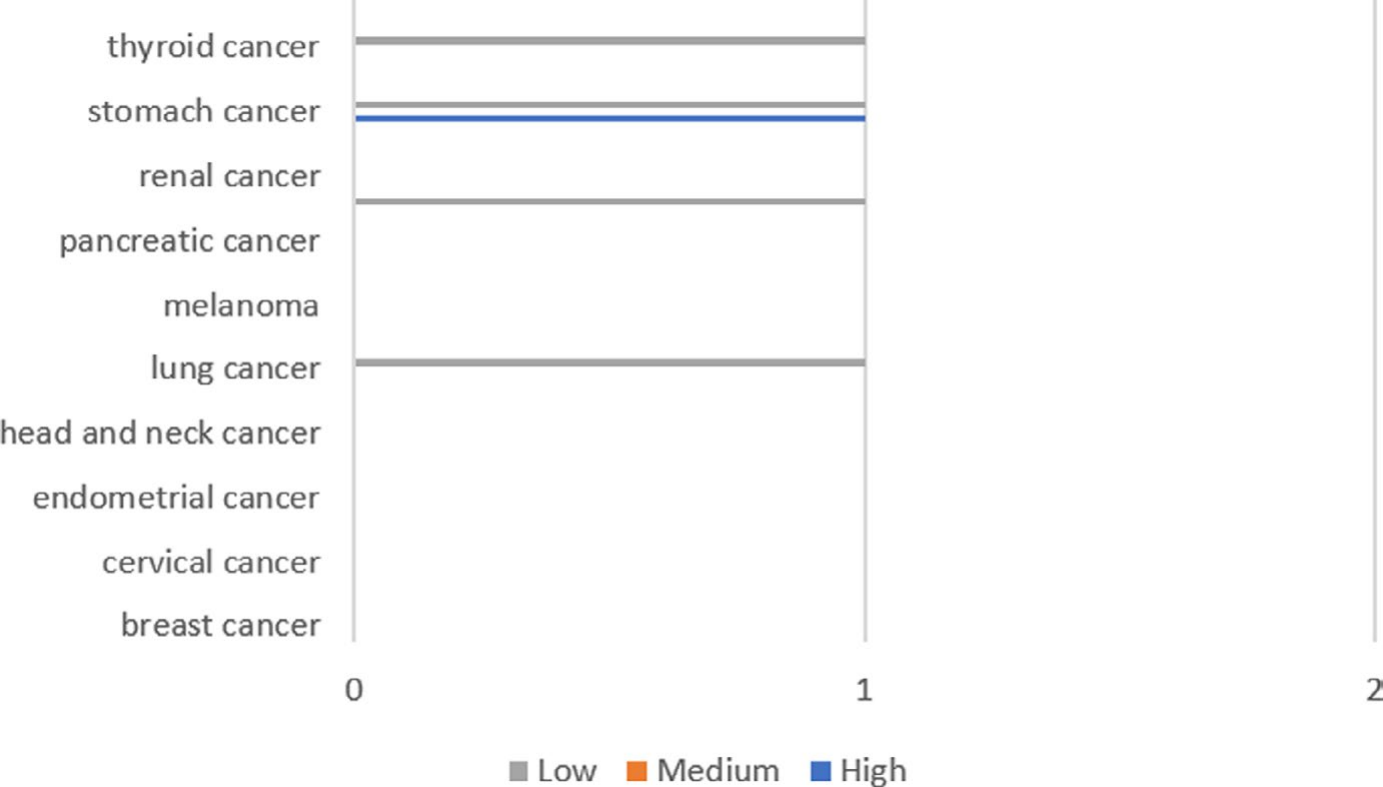
Expression of PGC protein in tumor tissues

**Figure 3:**
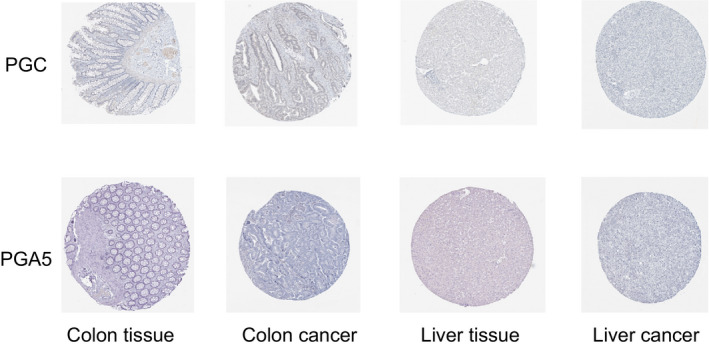
Expression of PGC and PGA5 proteins in different tissues

**Figure 4:**
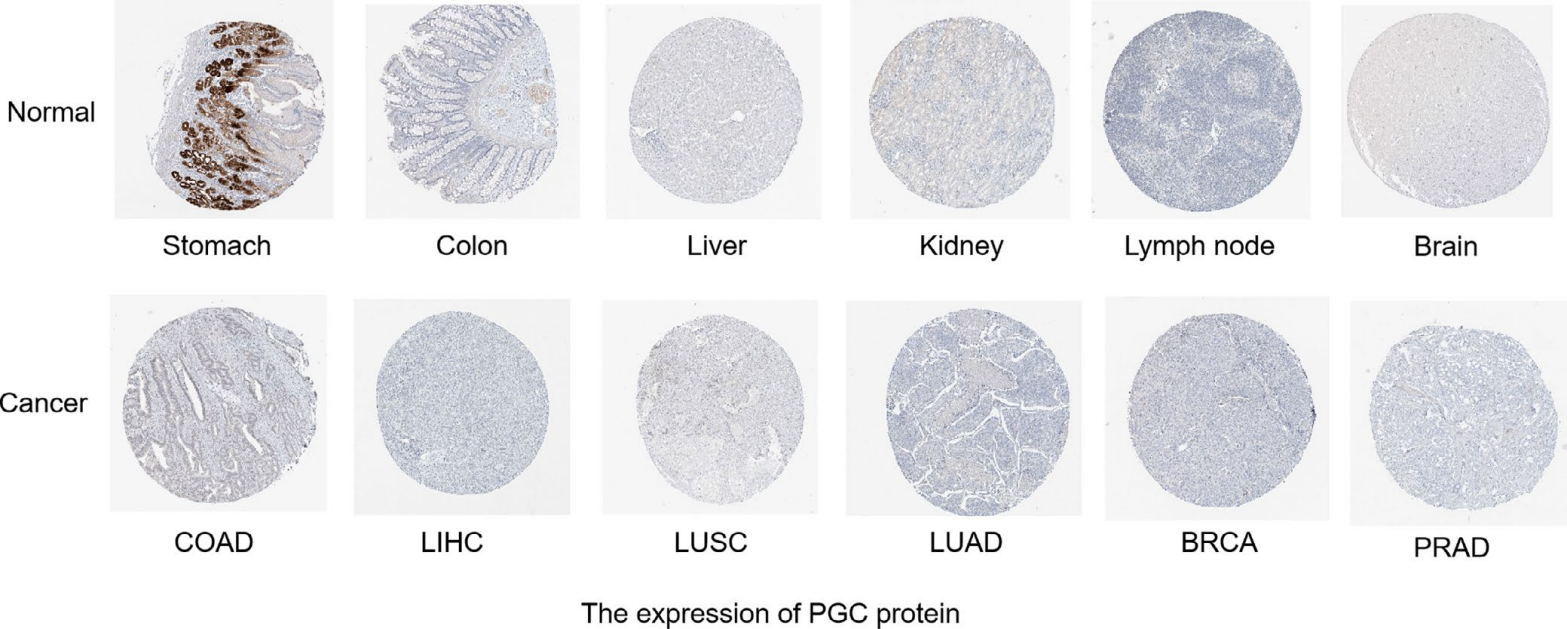
Expression of PGC protein in different human tissues

The data of 21 kinds of cancers from The Protein Atlas showed that the expression of PGA3, PGA4, and PGA5 could not be detected in any cancer tissues. Only a small amount of PGC expression was detected in lung, prostate, gastric, and thyroid tissues (Figure 2). PGC protein expression was higher in lung adenocarcinoma, and prostate adenocarcinoma but lower in stomach adenocarcinoma; but in other tissues, PGA protein was not detected. Based on the immunohistochemical results of the protein map database, we showed the protein expression of PG gene in different cancer types (Figure 3). The immunohistochemical results representing the expression of PGC protein are shown in Figure 4.

#### 3.1.3 PGs expression at cell level

**Figure 5:**
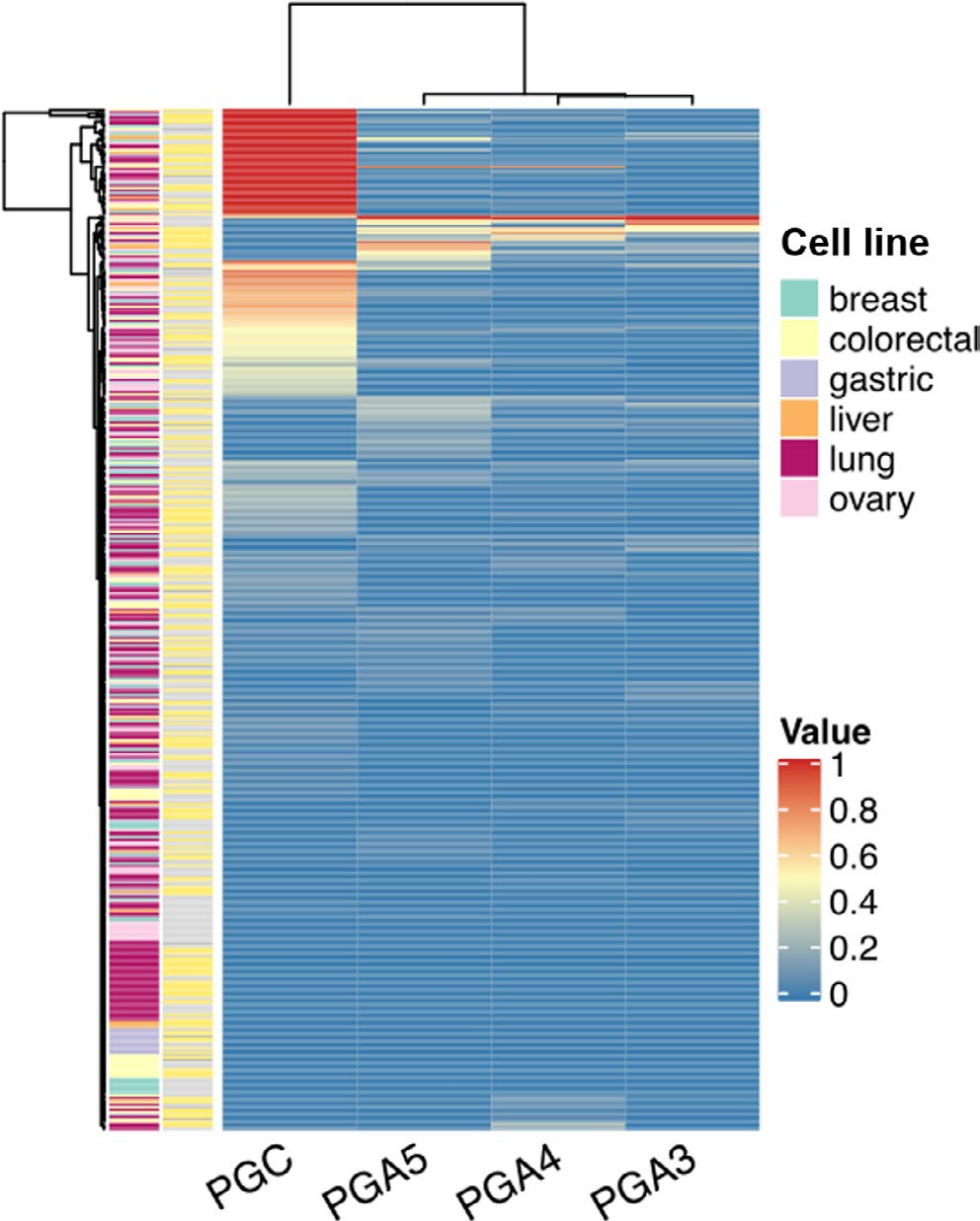
The PGs expression profile in each cell line in CCLE database

**Figure 6:**
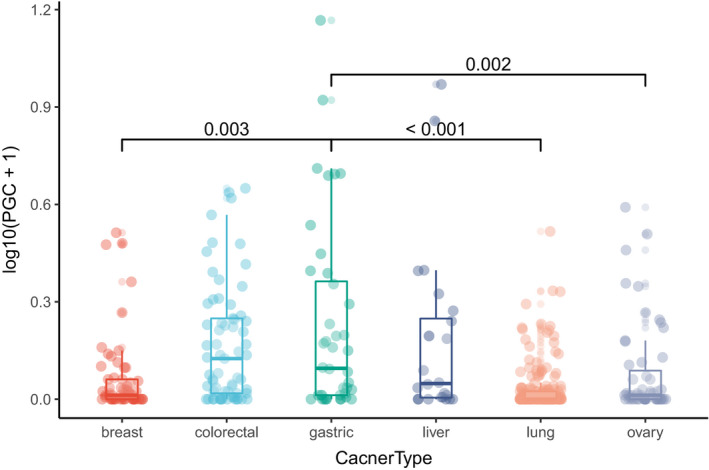
The expression of PGC gene in different cell lines in CCLE database

The CCLE analysis results showed that there was certain degree of PG expression in breast cancer, liver cancer, colorectal cancer, gastric cancer, ovarian cancer, and lung cancer cell lines (Figure 5). PGC was mainly expressed in gastric cancer, colorectal cancer, and liver cancer cell lines (Figure 6). PGA3 and PGA5 are mainly expressed in ovarian cancer and lung cancer, while PGA4 is mainly expressed in lung cancer and gastric cancer cell lines.

### 3.2 The activated transduction pathways related to PGs expression in pan‐cancer

**Figure 7:**
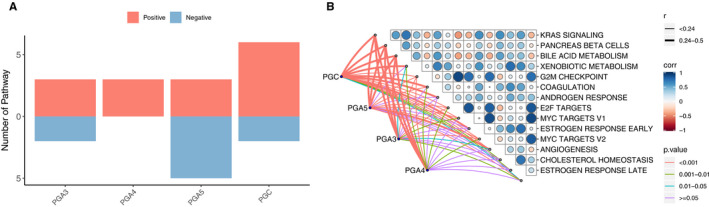
Association of PGs expression with cancer‐related pathways. A, The number of correlated pathways in each individual PGs. B, Correlation between the expression of different PGs and cancer‐related pathways

To clarify the molecular significance of PG gene family in tumorigenesis, we analyzed and visualized the relationship between the PGs expression and cancer‐related transduction pathway. The results show that PGs expression was significantly related to the activation or inhibition of many carcinogenic pathways (Figure 7A), in which PGC and PGA5 are more likely to be associated with carcinogenic processes. PGC was mainly involved in K‐RAS signaling pathway, bile acid metabolism, androgen response, estrogenic response, blood coagulation, and angiogenesis. PGA3, PGA4, and PGA5 are mainly involved in K‐RAS signaling pathway, bile acid metabolism, mitotic G2 M phase, and other cancer‐related pathways. PGA5 not only participates in the above pathways, but also participates in the mTOR pathway and DNA repair. The specific degree of correlation between the PGs expression and each cancer‐related pathway is summarized in Figure 7B. The correlation coefficient between PGs and cancer‐related pathways is shown in Table 1.

**Table 1:** Correlation between PGs expression and signal transduction pathways

Pathway	Correlation coefficient			
	PGC	PGA5	PGA3	PGA4
HALLMARK_ADIPOGENESIS	0.104074	0.072013	0.059938	0.088963
HALLMARK_ALLOGRAFT_REJECTION	0.073248	0.046939	0.082432	0.13315
HALLMARK_ANDROGEN_RESPONSE	0.258346	0.065494	0.124837	0.094096
HALLMARK_ANGIOGENESIS	0.232618	0.249746	0.189061	0.165817
HALLMARK_APICAL_JUNCTION	0.121867	0.146654	0.091354	0.13103
HALLMARK_APICAL_SURFACE	0.179791	0.218459	0.173584	0.184061
HALLMARK_APOPTOSIS	0.047602	0.045279	0.066674	0.082117
HALLMARK_BILE_ACID_METABOLISM	0.307126	0.260051	0.25485	0.253188
HALLMARK_CHOLESTEROL_HOMEOSTASIS	0.213147	0.131716	0.098256	0.175383
HALLMARK_COAGULATION	0.259254	0.239252	0.161715	0.217216
HALLMARK_COMPLEMENT	0.095378	0.13422	0.116431	0.12946
HALLMARK_DNA_REPAIR	−0.16514	−0.20049	−0.16615	−0.09401
HALLMARK_E2F_TARGETS	−0.25135	−0.29814	−0.24509	−0.21817
HALLMARK_EPITHELIAL_MESENCHYMAL_TRANSITION	−0.00166	0.061258	0.010087	0.019819
HALLMARK_ESTROGEN_RESPONSE_EARLY	0.242907	0.166006	0.150116	0.166
HALLMARK_ESTROGEN_RESPONSE_LATE	0.213013	0.153224	0.105702	0.145089
HALLMARK_FATTY_ACID_METABOLISM	0.111987	0.06063	0.081834	0.104381
HALLMARK_G2M_CHECKPOINT	−0.26701	−0.30841	−0.2584	−0.23313
HALLMARK_GLYCOLYSIS	0.134493	0.016814	0.040321	0.06998
HALLMARK_HEDGEHOG_SIGNALING	0.120912	0.132243	0.07334	0.102322
HALLMARK_HEME_METABOLISM	0.074388	0.111684	0.093911	0.095899
HALLMARK_HYPOXIA	0.067271	0.12587	0.087012	0.079801
HALLMARK_IL2_STAT5_SIGNALING	0.137145	0.13121	0.123792	0.131418
HALLMARK_IL6_JAK_STAT3_SIGNALING	0.176787	0.183303	0.135724	0.149932
HALLMARK_INFLAMMATORY_RESPONSE	0.138655	0.139786	0.135577	0.134099
HALLMARK_INTERFERON_ALPHA_RESPONSE	0.044547	−0.02577	0.013675	0.027553
HALLMARK_INTERFERON_GAMMA_RESPONSE	0.069933	0.034519	0.069113	0.105927
HALLMARK_KRAS_SIGNALING_DN	0.339758	0.34074	0.289109	0.322171
HALLMARK_KRAS_SIGNALING_UP	0.1654	0.146907	0.163549	0.169164
HALLMARK_MITOTIC_SPINDLE	−0.09698	−0.14517	−0.15048	−0.14056
HALLMARK_MTORC1_SIGNALING	−0.18071	−0.25302	−0.1992	−0.14975
HALLMARK_MYC_TARGETS_V1	−0.24327	−0.26186	−0.19809	−0.16793
HALLMARK_MYC_TARGETS_V2	−0.23571	−0.28542	−0.2638	−0.20025
HALLMARK_MYOGENESIS	0.148353	0.227962	0.158631	0.201877
HALLMARK_NOTCH_SIGNALING	0.039809	0.023217	0.018902	0.039152
HALLMARK_OXIDATIVE_PHOSPHORYLATION	0.01812	−0.01984	0.030688	0.047726
HALLMARK_P53_PATHWAY	−0.03243	−0.08171	−0.04491	−0.03136
HALLMARK_PANCREAS_BETA_CELLS	0.320524	0.303613	0.340141	0.296361
HALLMARK_PEROXISOME	−0.00907	−0.0782	−0.03897	0.00352
HALLMARK_PI3K_AKT_MTOR_SIGNALING	−0.04893	−0.03759	−0.00983	−0.00939
HALLMARK_PROTEIN_SECRETION	0.126021	−0.0117	0.009543	0.020547
HALLMARK_REACTIVE_OXYGEN_SPECIES_PATHWAY	0.065843	0.021639	0.049905	0.098622
HALLMARK_SPERMATOGENESIS	−0.01038	0.044703	0.057758	0.047252
HALLMARK_TGF_BETA_SIGNALING	−0.08331	−0.11723	−0.11123	−0.11492
HALLMARK_TNFA_SIGNALING_VIA_NFKB	0.097993	0.10124	0.095539	0.103767
HALLMARK_UNFOLDED_PROTEIN_RESPONSE	−0.08318	−0.16531	−0.11658	−0.08618
HALLMARK_UV_RESPONSE_DN	0.111441	0.063833	0.042253	0.03483
HALLMARK_UV_RESPONSE_UP	−0.00555	−0.06643	−0.05887	−0.03026
HALLMARK_WNT_BETA_CATENIN_SIGNALING	0.06264	0.012163	0.004281	0.0549
HALLMARK_XENOBIOTIC_METABOLISM	0.289237	0.173161	0.191408	0.222242

The above results suggested that PGC was mainly involved in cancer‐related pathways. Therefore, we further analyzed and visualized the relationship between PGC and cancer‐related pathways in order to further clarify the molecular significance of PGC gene in pan‐tumorigenesis. The results showed that PGC participated in 33 regulatory network pathways in pan‐cancer (*p* < 0.05, R > 0.24), mainly distributed in stomach adenocarcinoma, esophageal carcinoma, and lung squamous cell carcinoma, respectively. The stomach adenocarcinoma‐related pathways involved by PGC mainly include K‐RAS signal pathway, bile acid metabolism pathway, androgen response, blood coagulation process, estrogen response, and so on. The esophageal carcinoma‐related pathways included K‐RAS signal pathway, DNA repair, p53 pathway, protein secretion, TGF β signal pathway, WNT‐ β catenin signal pathway, and so on. The LUSC‐related pathways included DNA repair, IL6‐JAK‐STAT3 signaling pathway, inflammation, p53 pathway, and oxidative phosphorylation. The distribution and correlation degree of all cancer‐related pathways associated with PGC are shown in Table 2.

**Table 2:** Correlation between PGC expression and cancer‐related pathways

Pathway	Cancer type	R	*p* value
ANDROGEN_RESPONSE	STAD	0.258346	5.26E‐07
BILE_ACID_METABOLISM	STAD	0.307126	4.74E‐08
COAGULATION	STAD	0.259254	2.06E‐05
E2F_TARGETS	STAD	−0.25135	4.94E‐05
ESTROGEN_RESPONSE_EARLY	STAD	0.242907	0.000123
G2M_CHECKPOINT	STAD	−0.26701	8.43E‐06
KRAS_SIGNALING_DN	STAD	0.339758	3.70E‐10
MYC_TARGETS_V1	STAD	−0.24327	0.000119
PANCREAS_BETA_CELLS	STAD	0.320524	6.95E‐09
XENOBIOTIC_METABOLISM	STAD	0.289237	5.26E‐07
ADIPOGENESIS	ESCA	−0.29456	0.00255
APOPTOSIS	ESCA	−0.34617	0.000126
CHOLESTEROL_HOMEOSTASIS	ESCA	−0.2528	0.021382
DNA_REPAIR	ESCA	−0.34703	0.000122
E2F_TARGETS	ESCA	−0.28764	0.003607
G2M_CHECKPOINT	ESCA	−0.30326	0.001699
HEME_METABOLISM	ESCA	−0.35708	6.24E‐05
KRAS_SIGNALING_DN	ESCA	0.33191	0.000318
MITOTIC_SPINDLE	ESCA	−0.36193	4.47E‐05
MTORC1_SIGNALING	ESCA	−0.30286	0.001699
MYC_TARGETS_V1	ESCA	−0.31736	0.000754
MYC_TARGETS_V2	ESCA	−0.29547	0.002536
NOTCH_SIGNALING	ESCA	−0.24232	0.032938
OXIDATIVE_PHOSPHORYLATION	ESCA	−0.29483	0.00255
P53_PATHWAY	ESCA	−0.37275	2.07E‐05
PANCREAS_BETA_CELLS	ESCA	0.306135	0.00147
PEROXISOME	ESCA	−0.36227	4.46E‐05
PI3K_AKT_MTOR_SIGNALING	ESCA	−0.33754	0.000223
PROTEIN_SECRETION	ESCA	−0.32454	0.00049
SPERMATOGENESIS	ESCA	−0.25025	0.023348
TGF_BETA_SIGNALING	ESCA	−0.35218	8.67E‐05
UNFOLDED_PROTEIN_RESPONSE	ESCA	−0.32859	0.000386
WNT_BETA_CATENIN_SIGNALING	ESCA	−0.27507	0.006988
ANGIOGENESIS	LUSC	0.363518	5.68E‐17
DNA_REPAIR	LUSC	−0.33142	5.92E‐14
E2F_TARGETS	LUSC	−0.33102	6.28E‐14
G2M_CHECKPOINT	LUSC	−0.3295	8.38E‐14
GLYCOLYSIS	LUSC	−0.37583	3.19E‐18
IL6_JAK_STAT3_SIGNALING	LUSC	0.254868	4.38E‐08
INFLAMMATORY_RESPONSE	LUSC	0.307018	6.68E‐12
MTORC1_SIGNALING	LUSC	−0.3451	3.41E‐15
MYC_TARGETS_V1	LUSC	−0.34399	4.24E‐15
MYC_TARGETS_V2	LUSC	−0.34571	3.06E‐15
MYOGENESIS	LUSC	0.324046	2.48E‐13
OXIDATIVE_PHOSPHORYLATION	LUSC	−0.2636	1.16E‐08
P53_PATHWAY	LUSC	−0.27201	3.06E‐09
PEROXISOME	LUSC	−0.29628	4.75E‐11
UNFOLDED_PROTEIN_RESPONSE	LUSC	−0.36608	3.20E‐17

### 3.3 Correlation of PGs expression with tumor immune cells infiltration in pan‐cancer

**Figure 8:**
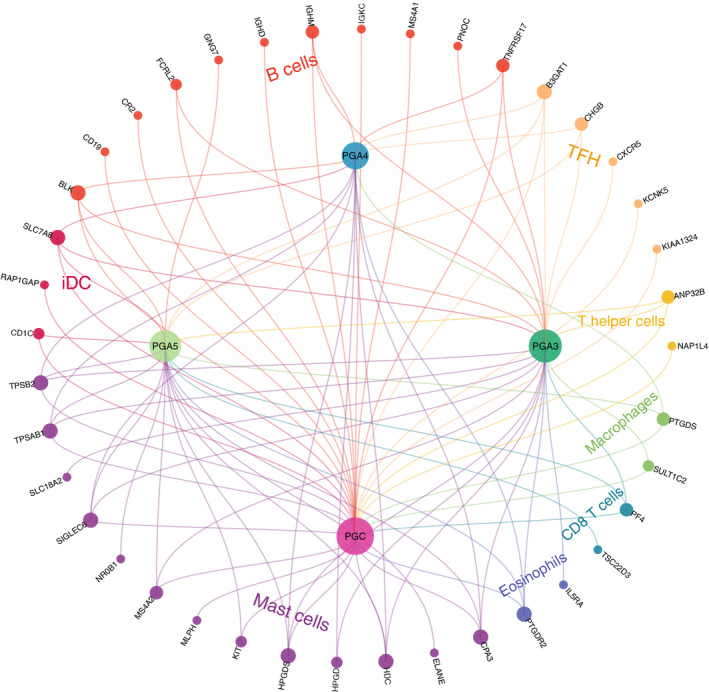
Correlation between PGs expression and immune cells infiltration. The genes in the outer circle represent genes within individual immune cells. Inner circles are formed by PGs. The size of each gene represents the number of connections

In this study, we explored the relationship between PGs expression and immune cells infiltration in pan‐cancer. The results showed that the immune cells most related to PGs included B cells, mast cells, follicular helper T cells (TFH cells), and helper T cells. PGC and PGA3 were significantly correlated with immune cell infiltration (Figure 8). The coefficient of the correlation between PGs and immune cell infiltration showed that the immune genes related to the PGs are PTGDR2, SULT1C2, HDC, HPGDS, B3GAT1, and TPSAB1 (Table S4).

### 3.4 Correlation between PGs expression and prognosis in pan‐cancer

**Figure 9:**
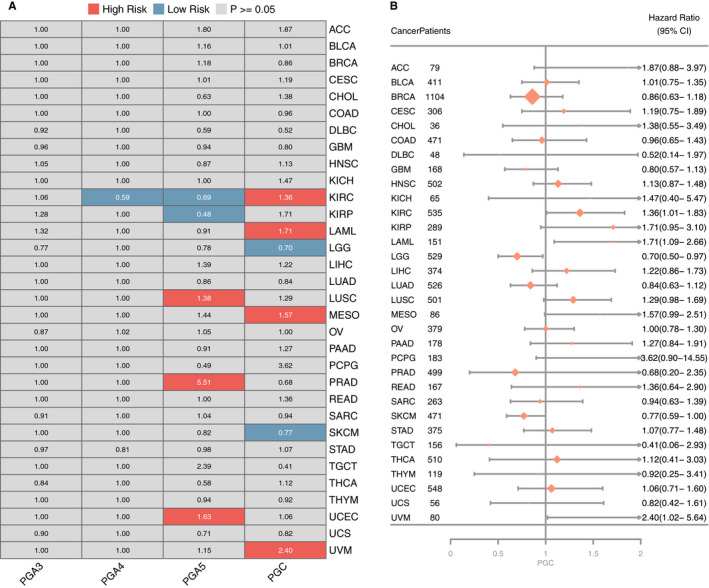
Prognostic significance associated with PGs expression. A, The correlation between PGs expression and cancer survival. Red color represents high risk of death while blue color represents low risk of death. B, Forest plot for the prognostic analysis of PGC across various cancer types

The prognostic significance of PGs in different cancer were analyzed by cox regression. The results showed that PGC was associated with higher survival rate in brain lower grade glioma, skin cutaneous melanoma, and poor survival rate in kidney renal clear cell carcinoma, acute myeloid leukemia, mesothelioma, and uveal melanoma. PGA4 is only associated with higher survival rate in kidney renal clear cell carcinoma. PGA5 is related to the prolongation of survival time of cancer patients in kidney renal clear cell carcinoma and kidney renal papillary cell carcinoma, while it is related to the shortening of survival time of cancer patients in lung squamous cell carcinoma, prostate adenocarcinoma, and uterine corpus endometrial carcinoma. Different PGs expression play significantly different prognostic roles in different types of cancer (Figure 9A). Furthermore, we drew a forest map in which PGC showed different prognostic correlations in different types of cancers (Figure 9B).

### 3.5 Characteristics of genetic variation of PGs in pan‐cancer

**Figure 10:**
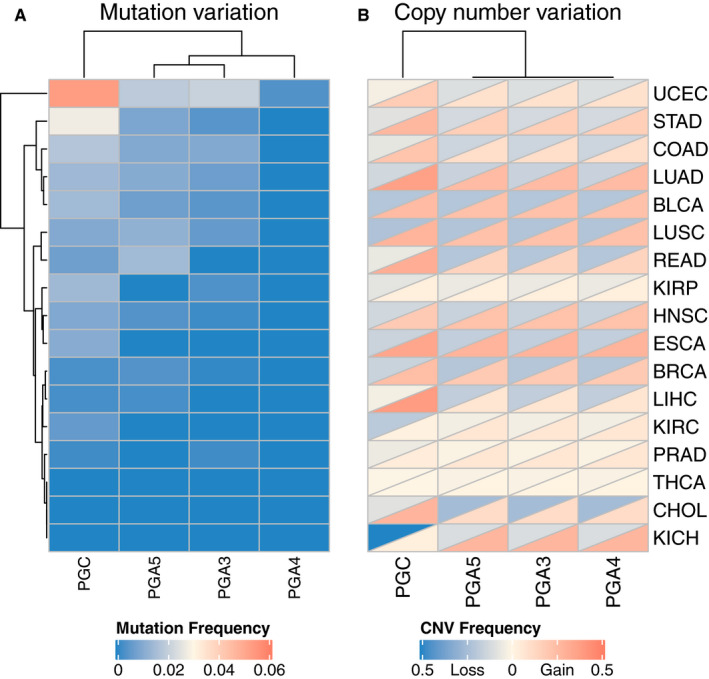
A, Mutation frequency of PGs in different cancers. B, The copy number variations frequency of PGs in different cancers

Using the TCGA database, we analyzed the mutation frequency of PGs. The results showed that PGC gene mutations frequently occurred in uterine corpus endometrial carcinoma and stomach adenocarcinoma (Figure 10A). The overall average mutation rate is 0‐5.3%. The mutation rates of PGA3, PGA4, and PGA5 in all cancer lines were low and less than 2%. In addition, we also analyzed the copy number variation （CNV）of PGs in different cancer cells (Figure 10B), PGC gene showed extensive copy number amplification in various cancer cells and decreased copy number only in kidney chromophobe. PGA3, PGA4, and PGA5 showed more copy number amplification in lung adenocarcinoma, esophageal carcinoma, kidney chromophobe, and copy number reduction in bladder urothelial carcinoma, lung squamous cell carcinoma, rectum adenocarcinoma, and cholangiocarcinoma.

**Figure 11:**
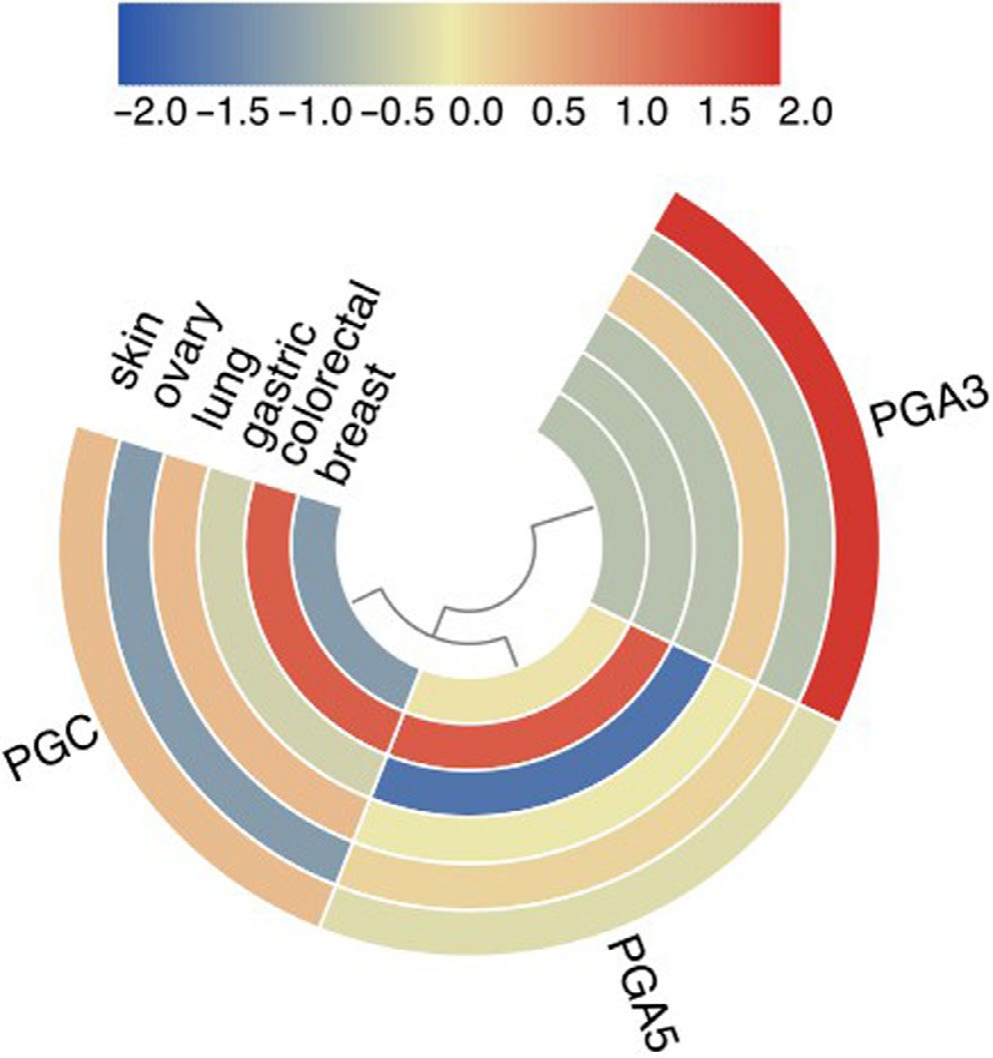
The mutation frequency of PGs across common cancer cell lines. Each circle from the outside to the inside represents a type of tumor cell line (breast, gastric, colorectal, kidney, lung, bone, ovary, skin, fibroblast, and liver)

In addition, CCLE database analysis revealed the mutation status of PGs in different human cancer cell lines, which showed that there were frequent mutations of PGs in colorectal cancer and gastric cancer cell lines (Figure 11).

### 3.6 Correlation between PGs mutation, copy number variation, and PGs expression

In order to explore whether PGC self‐variation affects its expression, we analyzed the correlation between PGs mutation, CNV, and PGs expression. The results showed that PGs mutations did not affect the PGs expression in all cancers. Except for PGC, the CNV of PGA3, PGA4, and PGA5 had no effect on their gene expression. PGC expression was upregulated with the increase of copy number in cholangiocarcinoma, esophageal carcinoma, and kidney renal papillary cell carcinoma, while in stomach adenocarcinoma, PGC was upregulated regardless of whether the copy number was increased or decreased. The effect of copy number of PGC gene on the PGC expression is shown in Table 3.

**Table 3:** Correlation between PGC CNV and PGC expression.

Cancer Type	CNV	n	Expression median value	*p* value
CHOL	DEL	3	0.003 (0.002‐0.089)	**0.022**
	GAIN	10	0.048 (0.016‐0.484)	
	No Change	23	0.006 (0.002‐0.017)	
COAD	DEL	31	0.017 (0.002‐0.211)	0.383
	GAIN	96	0.025 (0.008‐0.102)	
	No Change	321	0.018 (0.005‐0.086)	
DLBC	DEL	3	0.014 (0.008‐0.015)	0.067
	GAIN	10	0.001 (0.001‐0.002)	
	No Change	35	0.001 (0‐0.002)	
ESCA	DEL	25	0.004 (0‐0.83)	**0.017**
	GAIN	56	0.305 (0.004‐115.695)	
	No Change	79	0.027 (0.001‐5.911)	
KICH	DEL	50	0.002 (0.001‐0.003)	0.759
	GAIN	2	0.018 (0.009‐0.027)	
	No Change	13	0.003 (0‐0.008)	
KIRP	DEL	22	0.004 (0.001‐0.043)	**<0.001**
	GAIN	9	0.039 (0.025‐0.181)	
	No Change	255	0.001 (0‐0.003)	
LAML	GAIN	1	0.012 (0.012‐0.012)	0.238
	No Change	143	0.003 (0.001‐0.008)	
LIHC	DEL	10	0.011 (0.001‐0.549)	0.078
	GAIN	142	0.021 (0.001‐0.485)	
	No Change	212	0.002 (0‐0.198)	
LUAD	DEL	66	2.138 (0.194‐20.508)	**0.016**
	GAIN	185	8.301 (0.666‐211.746)	
	No Change	259	8.296 (0.587‐117.217)	
LUSC	DEL	109	0.182 (0.005‐1.905)	0.496
	GAIN	139	0.221 (0.007‐2.326)	
	No Change	250	0.34 (0.017‐2.256)	
PRAD	DEL	25	1.744 (0.288‐16.542)	0.104
	GAIN	21	21.602 (0.506‐43.134)	
	No Change	443	6.554 (1.374‐24.64)	
READ	DEL	10	0.012 (0.004‐0.045)	0.184
	GAIN	51	0.045 (0.013‐0.119)	
	No Change	103	0.028 (0.009‐0.121)	
STAD	DEL	34	47.186 (2.106‐647.67)	**0.032**
	GAIN	101	67.936 (2.409‐1399.045)	**0.020**
	No Change	239	14.919 (0.448‐530.709)	

Bold values represents statistical significance when p is less than 0.05.

## 4 DISCUSSION

In this study, we used the multilevel data of TCGA, Oncomine, and CCLE to reveal the expression and activated pathways, mutation, and copy number variation, prognostic potential of PGs in all 33 types of tumors and 431 cell lines, aiming to clarify the important role of PGs in tumorigenesis and development of cancers. The results suggest that there was differential expression of PGs between many kinds of cancer tissues and corresponding normal tissues, which is related to the prognosis of patients; PGs expression was closely associated with the activation of cancer‐related pathways and immune cell infiltration; the copy number variation of PGC could affect the gene expression. To our knowledge, this study first drew a panoramic picture of pepsinogen gene family in human cancer.

In this study, a multidimensional analysis of PGs expression in pan‐cancer based on TCGA data was conducted at mRNA, protein, and cell level, respectively. The mRNA expression of PGs was detected in 16 of all 33 kinds of tumors, while PGs was not detected in another 17 tumors. Among the 16 cancers with PGs positive expression, PGs was unevenly expressed with different levels in different cancers (Figure 1). At the protein expression level, only a small amount of PGC expression was detected in lung cancer, prostate cancer, and thyroid carcinoma, but no PGA expression was detected. At the cell detection level, there was certain degree of PGs expression in breast cancer, liver cancer, colorectal cancer, gastric cancer, ovarian cancer, and lung cancer cell lines. Validation results based on three well‐studied cancer types including stomach adenocarcinoma, lung squamous cell carcinoma, and colorectal adenocarcinoma from Oncomine database were consistent with our TCGA findings. By retrieving published literature, we also found supporting evidence that the expression of PGC in situ in gastric mucosa has a good correlation with the occurrence and development of stomach adenocarcinoma, and it is an ideal "negative marker" for stomach adenocarcinoma.^1^, ^5^, ^14^, ^15^ Beside, PGC also have close relationship with ovarian cancer,^16^ breast cancers,^10^, ^17^ and prostatic cancer.^8^, ^18^ PGA is expressed in esophageal squamous cell carcinoma.^11^ In this study, through pan‐cancer analysis, a panoramic view of PGs expression profile in all human cancers was first showed up and the results suggest that PGs, especially PGC, have characteristic of broad‐spectrum expression in multiple cancers, which may made PGs be useful biomarkers for prediction/diagnosis/prognosis and effective targets for treatment in human cancer, which is an interesting and new research topic in the relevant fields.

The correlation analysis between PGs expression and cancer signal transduction pathway showed that 50 cancer‐related pathways were associated with PGs expression in 33 cancers, such as K‐RAS signal pathway, bile acid metabolism pathway, amino acid metabolism pathway, androgen and estrogen response pathway, mitosis, DNA repair pathway and angiogenesis. Different PGs expression have been found to be associated with different cancer‐related pathways, indicating that different PGs in the same pepsinogen family have different functional effects. Among them, PGC and PGA5 are more likely to be related to the carcinogenic process. It is reported that PGC was highly expressed in breast cancer.^19^ The results of this study show that PGC was closely related to androgen response pathway and estrogen response pathway, suggesting that PGC participates in hormone‐related pathways and plays a regulatory role in the occurrence and development of breast cancer. In addition, it is of particular note that PGC was involved in 33 signal pathways, mainly in three cancers including stomach adenocarcinoma, esophageal carcinoma and lung squamous cell carcinoma. According to the literature, the synthesis of pepsin can happen in Barrett's esophagus and early esophageal carcinoma. The loss of pepsinogen in advanced esophageal squamous cell carcinoma indicates that pepsin is involved in the process of protein synthesis in the esophagus and causes esophageal carcinogenesis.^20^, ^21^ The results of this study further confirmed that PGC is involved in the regulation of esophageal carcinoma. Both lung tissue and gastric mucosa have the same function of producing pepsinogen molecules,^11^ and the injury of normal lung tissue could increase the synthesis of pepsinogen C.^22^ Some studies have also suggested that the existence of pepsin in respiratory biological samples was caused by gastroesophageal reflux associated lung inhalation.^23^ Another study has been reported that there was a certain degree of PG expression in lung type 2 epithelial cells.^24^ The results of this study showed that the activation pathways with PGs expression is associated with the lung squamous cell carcinoma. Further research should more accurately evaluate the expression of pepsinogen and its dynamic changes in the occurrence and development of lung squamous cell carcinoma. All in all, the results mentioned above indicate that PGs family, especially PGC, may participate in the signal transduction pathways during the occurrence and development of multiple cancers and may play a synergistic role in the process.

The correlation analysis between PGs expression and tumor immune cell infiltration in pan‐cancer showed that the immune infiltrating cells related to PGs included B cells, mast cells, TFH cells, and helper T cells. At present, there are few studies on the relationship between PGs and immune cell infiltration. Al‐Ezzy et al found that the secretion of PGA and PGC was related to the immune response of *Helicobacter pylori* infection.^25^ Matveeva et al. reported that serum PGA and PGC levels of gastric ulcer patients were significantly increased along with changes of macrophages and cell‐humoral balance.^26^ Animal experiments had shown that pepsin and pepsinogen are abundant in immune cells and plasma, and the production of interleukin‐1 in vivo may be partially regulated by the plasma concentration of pepsin and pepsinogen.^27^ Hara et al. revealed that pepsinogen can bind nonspecifically to immune complexes and immunoglobulins.^28^ Combined the previous research with the results of our study, it is not hard to see that there is close relationship between PGs and tumor immune cell infiltration, which may provide a new idea for the research of tumor immunotherapy targeted PGs in the future.

The correlation analysis between PGs expression and prognosis in pan‐cancer showed PGC was correlated with high survival rate of cancer patients in kidney renal clear cell carcinoma, acute myeloid leukemia, mesothelioma, and uveal melanoma. PGA5 was associated with good prognosis of cancer patients such as kidney renal clear cell carcinoma and kidney renal papillary cell carcinoma, while lung squamous cell carcinoma, prostate adenocarcinoma, and endometrial carcinoma are associated with poor prognosis of cancer patients. Previous studies have shown that PGC expression and tumor size are independent prognostic factors for overall survival and disease‐free survival in hepatocellular carcinoma.^29^ PGC was also an important prognostic factor in predicting longer survival of patients with prostate adenocarcinoma.^30^ Our findings suggest that PGC and PGA5 had different effects on the prognosis of many kinds of cancers and they may be used as predictors of the prognosis in different cancers. It is worth pointing out that cancer prognosis was affected by many factors. In addition to consider the characteristics of the cancer itself, the role of the local microenvironment of the organs and tissues also should pay more attention, in which the cancer occurs. The balance of "seed and soil" determined the outcome of the cancer. The different prognostic role of PGs in different cancer indicates that it could play different potential in different tumor microenvironment. It is necessary to further explore the internal molecular mechanism of organ‐specific prognostic role of PGs.

In our study, not only the parameters related to PGs expression were analyzed, but also the mutation and CNV of PGs were analyzed. The results showed that the overall average mutation rate of PGs was 0%‐5.3%, and the mutation rate of PGC was higher in stomach adenocarcinoma and endometrial carcinoma. It is worth noticed that all PGC, PGA3, and PGA5 genes had a certain degree of mutation in endometrial carcinoma, which is a tumor with high global mutation rate.^31^ In addition, CCLE‐based analysis of human cancer cell lines showed that most of the PGs mutations were found in colorectal adenocarcinoma and stomach adenocarcinoma cell lines, suggesting PGs mutation may be the key events in tumorigenesis and development of both gastric cancer and colorectal adenocarcinoma. In this study, we also found that there was extensive copy number amplification in various tumor types, which may be related to its widespread expression in various tissues. Furthermore, the effects of PGs mutation and CNV on PGs expression were analyzed in order to understand the influence of PGs inherent regulatory mechanism on PGs expression. The results showed that there was no correlation between PGs mutation and PGs expression in cancer cells. However, previous studies in our lab have found that PGC gene insertion‐deletion fragment polymorphism and single nucleotide polymorphism from human germline cells can affect PGC expression.^32^ Both somatic gene mutations and germline cell polymorphisms are often base variation in DNA sequences, and key variation in gene structure often lead to changes in gene expression. Therefore, the findings of this study need to be further verified. In addition, we also analyzed the effect of PGs CNV on the gene expression and found that there was no correlation between PGA CNV and expression. In cholangiocarcinoma, esophageal cancer, and kidney renal papillary cell carcinoma, PGC expression was upregulated with the increase of copy number, but in stomach adenocarcinoma, both increase and deletion of PGC copy number could lead to the up‐regulation of PGC expression. Studies have shown that the increase in copy number was often matched with the upregulation of expression, but there are also some complex regulatory mechanisms existed, which make the correlation between CNV and gene expression very weak, sometimes even on the contrary.^33^ In some cancers, there may be transcriptional and posttranscriptional regulation, resulting in inconsistent changes in copy number and expression. For genes with increased copy number and decreased expression, there may be related noncoding RNA regulation to inhibit gene expression caused by CNV amplification.^34^ The mechanism of inhibition of expression needs to be further explored and verified, so that we will have a better understanding of CNV inhibition and enhanced bi‐directional switching. In brief, genetic changes played a crucial part in the regulation of PGC expression. Its regulatory mechanism on the expression is worthy of our further study and exploration.

In conclusion, our study systematically demonstrated the expression profile of PG gene family as well as their activation pathways involved in human cancer. The relationship between PGs expression and clinical phenotypic characteristics was also explored from multi‐angle. Moreover, the genetic variations of PGs own structure and their internal effects on the PGs expression was further elucidated. We found that PGs was expressed unevenly in a variety of cancer tissues and was related to many carcinogenic pathways and involved in the immune regulation. PGC participated in 33 regulatory pathways in pan‐cancer. Different PGs expression play significantly different prognostic roles in different cancers. The variation of copy number of PGC gene could affect the PGC expression. These findings suggested that PGs, especially PGC have characteristic of broad‐spectrum expression in multiple cancers rather than being confined to the gastric mucosa, which may made PGs be useful biomarkers for prediction/diagnosis/prognosis and effective targets for treatment in human cancer. Our study provides detailed and accurate analysis data for in‐depth understanding of the relationship between PGs expression and phenotypic characteristics in human cancer and provide new clues for accurate diagnosis and treatment of PGs‐target cancers.

## CONFLICT OF INTEREST

The authors declare that they have no conflict of interest.

## AUTHORS’ CONTRIBUTION

Conceived and designed the experiments: Yuan Yuan and Liping Sun. Analyzed the data: Shixuan Shen, Hao Li, and Jingwei Liu. Wrote the paper: Shixuan Shen. Revised the manuscript: Yuan Yuan.

## Supporting information

Fig S1

Table S1

Table S2

Table S3

Table S4

## Data Availability

All the data come from TCGA, CCLE, and Oncomine databases. Other people could use these data.
