# Supplementary figures and images for: The panoramic picture of pepsinogen gene family with pan‐cancer
Source: Cancer Med. 2020 Oct 17;9(23):9064–80. doi: 10.1002/cam4.3489 (PMC7724489; doi:10.1002/cam4.3489)

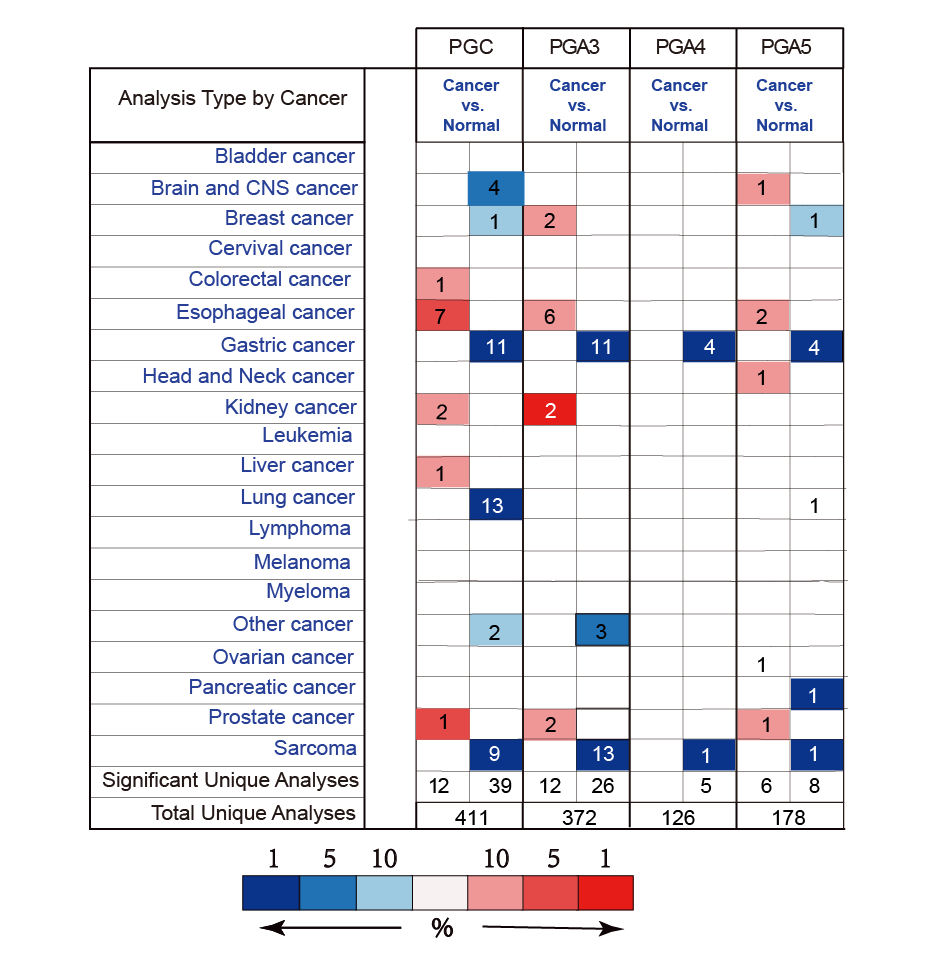

Supplement: Supplementary file 1 — Fig S1 [file CAM4-9-9064-s001.tif]
